# Potential roles of synaptotagmin family members in cancers: Recent advances and prospects

**DOI:** 10.3389/fmed.2022.968081

**Published:** 2022-08-08

**Authors:** Huandan Suo, Nan Xiao, Kewei Wang

**Affiliations:** ^1^Department of Surgery, The First Affiliated Hospital of China Medical University, Shenyang, China; ^2^Department of Gastrointestinal Surgery, The First Affiliated Hospital of China Medical University, Shenyang, China

**Keywords:** synaptotagmin, cancer, overexpression, biomarker, prognosis, oncogene

## Abstract

With the continuous development of bioinformatics and public database, more and more genes that play a role in cancers have been discovered. Synaptotagmins (SYTs) are abundant, evolutionarily conserved integral membrane proteins composed of a short N-terminus, a variable linker domain, a single transmembrane domain, and two C2 domains, and they constitute a family of 17 isoforms. The synaptotagmin family members are known to regulate calcium-dependent membrane fusion events. Some SYTs play roles in hormone secretion or neurotransmitter release or both, and much evidence supports SYTs as Ca^2+^ sensors of exocytosis. Since 5 years ago, an increasing number of studies have found that SYTs also played important roles in the occurrence and development of lung cancer, gastric cancer, colon cancer, and other cancers. Down-regulation of SYTs inhibited cell proliferation, migration, and invasion of cancer cells, but promoted cell apoptosis. Growth of peritoneal nodules is inhibited and survival is prolonged in mice administrated with siSYTs intraperitoneally. Therefore, most studies have found SYTs serve as an oncogene after overexpression and may become potential prognostic biomarkers for multiple cancers. This article provides an overview of recent studies that focus on SYT family members’ roles in cancers and highlights the advances that have been achieved.

## Introduction

**FIGURE 1:**
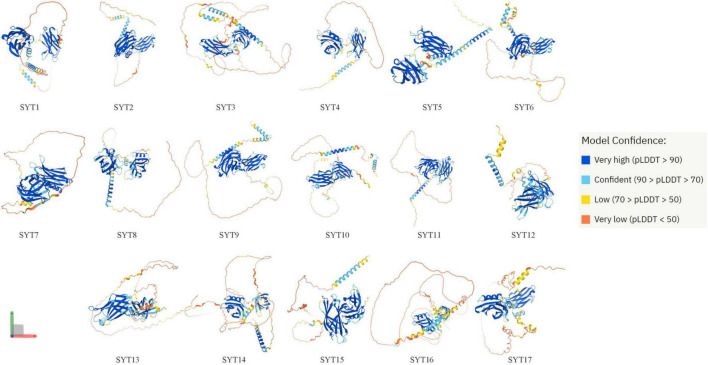
Structure of 17 human synaptotagmin family members predicted by AlphaFold. AlphaFold is an AI system developed by DeepMind that predicts a protein’s 3D structure from its amino acid sequence. AlphaFold produces a per-residue confidence score (pLDDT) between 0 and 100. Some regions below 50 pLDDT may be unstructured in isolation.

Synaptotagmins (SYTs) are a family of membrane-trafficking proteins composed of a short N-terminus, a variable linker domain, a single transmembrane domain, and two C2 domains (C2A and C2B domains) (1). The C2 domains are binding sites of Ca^2+^. SYT1–3, 5–7, and 9 have C2 domains, while the others do not (2). SYTs are known to regulate calcium-dependent membrane fusion events. At present, it has been found that humans have 17 synaptotagmin isoforms whose structure can be predicted by AlphaFold Protein Structure Database (3, 4) (Figure 1). The structure of some SYTs has been resolved for a long time (5–7). SYTs are involved in postsynaptic receptor endocytosis (8), synaptic vesicle exocytosis (9), synaptic plasticity (10), and vesicle trafficking (11). Since 5 years ago, an increasing number of studies have found that SYTs also play an important role in the occurrence and development of lung cancer, gastric cancer (GC), colon cancer, and other cancers (12–15) (Supplementary Figure 1). This article provides an overview of recent studies that focus on SYT family members’ roles in cancers and highlights the advances that have been achieved (Table 1).

**TABLE 1:** Potential functions of SYTs in cancers.

References	Isoform	Location	Prognostic predictors	*IN VIVO*	*IN VITRO*	Functions
Lu et al. (17)	SYT1	Colon cancer			√	Promote cell proliferation, invasion, and migration, inhibit apoptosis
Xiao et al. (27)	SYT7	Glioblastoma			√	Inhibit cellular apoptosis, promote cell growth
Kanda et al. (13)	SYT7	Gastric cancer	√		√	Promote cell proliferation, invasion, migration, and adhesion ability
				√		Increase hepatic metastasis
Wang et al. (15)	SYT7	Colorectal cancer			√	Promote cell proliferation and colony formation, inhibit G2/M arrest and apoptosis
Liu et al. (14)	SYT7	NSCLC			√	Promote cell proliferation, invasion, metastasis and EMT, inhibit apoptosis
				√		Promote growth of tumor
Fei et al. (26)	SYT7	Lung cancer			√	Inhibit cell senescence, promote growth and colony forming capacity
Wu et al. (28)	SYT7	Osteosarcoma			√	Promote cell proliferation, colony forming capacity, invasion and migration capability, inhibit apoptosis
Jin et al. (24)	SYT7	HCC	√		√	Promote cell proliferation and colony-forming ability
Fu et al. (29)	SYT7	HNSCC	√		√	Promote cell proliferation, inhibit apoptosis
				√		Promote migration and tumor growth
Kanda et al. (34)	SYT8	Gastric cancer			√	Promote cell invasion, migration, and fluorouracil resistance
				√		Promote the growth of peritoneal nodules, shorten survival time
Fu et al. (31)	SYT8	Pancreatic cancer		√	√	Promote cell proliferation, invasion and migration
Eizuka et al. (38)	SYT12	OSCC			√	Promote cell proliferation, invasion, and migration
Liu et al. (39)	SYT12	LUAD	√		√	Increase the proliferation and migration
				√		Increase the volume and weight of the tumors
Jin et al. (12)	SYT12	PTC			√	Promote cell colony formation, proliferation, invasion and migration, inhibit the process of apoptosis
Kanda et al. (42)	SYT13	Gastric cancer	√		√	Promote cell activity of invasion and migration, but did not alter proliferation and apoptosis
				√		Promote the growth of peritoneal nodules, shorten survival time
Zhang et al. (47)	SYT13	LUAD			√	Promote proliferation and clonal activity, inhibit apoptosis, increase migration capacity
Li et al. (45)	SYT13	Colorectal cancer			√	Promote cell proliferation, colony formation, invasion, migration and EMT
				√		Promote growth of tumor
Sheng et al. (51)	SYT14	Glioma			√	Promote cell proliferation and colony formation, inhibit apoptosis

NSCLC, non-small cell lung cancer; HCC, hepatocellular carcinoma; HNSCC, head and neck squamous cell carcinoma; OSCC, oral squamous cell carcinoma; LUAD, lung adenocarcinoma; PTC, papillary thyroid cancer.

## Roles of synaptotagmin family members in cancers

### SYT1

SYT1 is located at the vesicular membrane of nerve and endocrine cells. It is considered to be the main Ca^2+^ sensor in neurotransmission and hormone secretion processes and plays a vital role in Ca^2+^-induced secretion processes (16). In a recent study, downregulation of SYT1 significantly suppressed the proliferation, invasion, and migration of colon cancer cells, but induced cell apoptosis. These results suggested that SYT1 may serve as an oncogene in colon cancer (17).

### SYT7

SYT7 resides on human chromosome 11q12.2 and encodes a predicted single-pass 46-kDa transmembrane protein (18). SYT7 encodes a protein that plays a central role in the regulation of calcium-dependent lysosome exocytosis (19), facilitation of central synapses (20, 21), and the regulation of membrane trafficking during synaptic transmission (20, 22). SYT7 is currently the most studied isoform in oncological diseases.

Compared with adjacent normal tissues, SYT7 was found overexpressed in tissues of GC with hepatic metastasis. Meanwhile, high expression of SYT7 in primary GC tissues was closely correlated with hepatic recurrence, metastasis (stage IV GC), and adverse prognostic characteristics. Knockdown of SYT7 suppressed the proliferation of GC cells and attenuated the invasion, migration, and adhesion ability of cancer (13). It has also been reported that SYT7 is overexpressed in colorectal cancer (CRC). The higher level of SYT7 was significantly associated with a higher pathological stage of CRC. Downregulation of SYT7 inhibited RKO cell proliferation and colony formation but promoted G2/M arrest and subsequent apoptosis (15). Another study showed that SYT7 was significantly overexpressed in hepatocellular carcinoma (HCC) and was closely correlated with tumor size, differentiation, vascular invasion, and lymph node metastasis. Meanwhile, SYT7 was also identified as a risk factor for disease-free survival (DFS) and overall survival (OS). Additionally, knockdown of SYT7 in HCC could inhibit cell proliferation and colony-forming ability as well as induce cell cycle arrest (23, 24).

One recent study showed that SYT7 was upregulated in non-small-cell lung cancer (NSCLC), and its high expression was positively correlated with T stage and tumor differentiation. Patients with lower SYT7 expression had longer survival than those with higher expression (25). Liu et al. reported that SYT7 served as an oncogene in NSCLC *in vitro*, including promoting proliferation, invasion, and migration, but inhibiting apoptosis of cancer cells. It was also demonstrated that shSYT7 significantly blocked the growth of NSCLC tumor cells in a xenograft model. The expression of Vimentin and N-cadherin in cultured cells was decreased after the knockdown of SYT7, while E-cadherin levels increased (14). The above results revealed that SYT7 played a vital role in promoting tumorigenesis by activating epithelial-mesenchymal transition (EMT) in NSCLC. Another study demonstrated that the expression levels of SYT7 were elevated in both lung cancer tissues and cell lines. In addition, SYT7 was shown to inhibit senescence and promote growth and colony-forming capacity in lung cancer cells. The interaction between SYT7 and P53 further potentiated the interaction between P53 and its E3 ligase MDM2 (26).

Downregulation of SYT7 can promote cellular apoptosis and subsequently inhibit the growth of glioblastoma (27). One study by Wu et al. demonstrated that the expression levels of SYT7 in osteosarcoma tissues had a positive correlation with tumor stage. Functional assays showed that SYT7 silencing could significantly suppress cell proliferation as well as the colony-forming ability of osteosarcoma *in vitro* with time independence. Furthermore, knockdown of SYT7 could also increase cell apoptosis rates, induce cell cycle arrest with a decreased proportion of S phase and increased G2 phase, and inhibit the invasion and migration capability (28). Fu et al. discovered that downregulation of SYT7 obviously inhibited the migration and tumor growth of head and neck squamous cell carcinoma (HNSCC) *in vivo*. They also found that ΔNp63α could affect HNSCC cells by downregulating the expression of SYT7 *in vitro*, including inhibiting proliferation, promoting apoptosis, and reducing the proportion of cancer cells in G1 phase. Therefore, the ΔNp63α/SYT7 axis might be a potential clinically effective target for the treatment of HNSCC (29).

In summary, SYT7 may serve as an oncogene in GC, CRC, HCC, lung cancer, glioblastoma, osteosarcoma, and HNSCC. High expression of SYT7 predicted a poor prognosis in GC, HCC, and HNSCC. Nevertheless, the precise mechanisms underlying the biological activity of SYT7 remain to be elaborated on in these cancers.

### SYT8

There are two forms of SYT8 (40 and 50 kDa). The 40-kDa form was present in the cytosol in the brain, in clonal beta-cells, and in PC12 cells, while the 50-kDa form was localized in very typical clusters. Further research found that SYT8 is not a Ca^2+^ sensor in exocytotic membrane fusion in endocrine cells (30). There are few studies on SYT8 function; therefore, the possible functions of SYT8 in the brain or other organs are still unknown. However, in recent years, several studies have shown that abnormal expression of SYT8 may affect the occurrence and development of tumor diseases. SYT8 was overexpressed in tumor tissues of pancreatic cancer and played important roles in promoting cell proliferation, invasion, and migration both *in vivo* and *in vitro*. Furthermore, the authors also identified SYT8 to be involved in signaling *via* the TNNI2/ERRα/SIRT1 axis (31).

High expression scores of the dual-marker expression panel (MAGED2 and SYT8) were significantly correlated with higher tumor stage, more lymph node or peritoneal metastasis, and more vascular invasion. Moreover, compared with single markers, the C-index of the combination panel was obviously higher. Patients with GC can be precisely stratified into high, intermediate and low risk by this dual-marker predictive signature after gastrectomy (32). The authors also found that the optimal expression panel comprised four constituents (SYT8, MAGED2, FAM461, and BTG) among 32,767 combinations with a C-index value of 0.793. Both OS and DFS decreased incrementally with increasing expression scores (33). Kanda et al. found that the expression levels of SYT8 were higher in GC tissues of patients with peritoneal recurrence or metastasis. Downregulation of SYT8 in GC cells was correlated with inhibition of cell invasion, migration, and fluorouracil resistance. The growth of peritoneal nodules was significantly suppressed by intraperitoneal administration of SYT8-siRNA into nude mice engrafted with GC cells, and survival was also prolonged (34).

Finally, it may be concluded that SYT8 may serve as an oncogene in pancreatic cancer and GC. The underlying mechanism in pancreatic cancer could be involved in TNNI2/ERRα/SIRT1 signaling pathway.

### SYT12

SYT12 encodes proteins involved in regulating transmitter release in the nervous system (35). Moreover, SYT12 phosphorylation by cAMP-dependent protein kinase is essential for hippocampal mossy fiber long-term potentiation (36). In the field of oncology, a prospective cohort study investigated the effect of biomarkers (SYT12, ITGA2, and CDH3) on outcomes of papillary thyroid cancer (PTC) patients. SYT12 as a single marker provided the best prediction performance of initial metastasis (specificity: 54%; sensitivity: 72%) compared with ITGA2 and CDH3. For long-term outcomes, the best performance was obtained by combining American Thyroid Association risk stratification with SYT12, with a specificity and sensitivity of 73 and 88%, respectively (37). The above results suggest that SYT12 may serve as a prognostic biomarker in PTC but warrants to be further validation in larger populations. In The Cancer Genome Atlas (TCGA) cohort, SYT12 was significantly upregulated in PTC. Moreover, the expression levels of SYT12 were positively related to the incidence of lymph node metastasis. Functional experiments *in vitro* showed that knockdown of SYT12 inhibited cell colony formation, proliferation, migration, and invasion ability of PTC cell lines, but accelerated the process of apoptosis (12).

Eizuka et al. found that SYT12 was overexpressed in both oral squamous cell carcinoma (OSCC)-derived cell lines and primary OSCC tissues. Knockdown of SYT12 in OSCC inhibited cellular proliferation, invasion, and migration and arrested the cell cycle in G1 phase. Meanwhile, L-dopa (L-3,4-dihydroxyphenylalanine), which has been approved for Parkinson’s disease, could reduce cellular SYT12 expression, allowing cells to acquire a cellular phenotype similar to SYT12 knockdown. Therefore, L-dopa is expected to become a new drug for the clinical therapy of OSCC by regulating the expression level of SYT12 (38). Analysis through the public database TCGA database revealed that SYT12 expression was significantly increased in tissues of lung adenocarcinoma (LUAD). Moreover, SYT12 was confirmed to be correlated with advanced tumor stage and poor prognosis. SYT12 also promoted LUAD cell proliferation and migration *in vitro* and increased the weight and volume of tumors in mice xenograft models. In parallel, SYT12 could activate the PI3K/AKT/mTOR signaling pathway by increasing the level of phosphorylation of PIK3R3 (39).

To summarize, SYT12 may serve as an oncogene in PTC, OSCC, and LUAD. High expression of SYT12 predicted poor prognosis in LUAD. The underlying mechanism in LUAD could be involved in signaling *via* the PI3K/AKT/mTOR axis.

### SYT13

SYT13 locates at human chromosome 11p11.2 and encodes a predicted single-pass 47-kDa transmembrane protein (40). SYT13 also serves as a neuroendocrine marker in the pancreas, intestine, and brain (41). In an analysis of 200 GC patients, the expression levels of SYT13 mRNA in patients with peritoneal recurrence or metastasis were significantly higher than those in Stage I GC patients. SYT13 knockdown in a GC cell line obviously reduced cell migration and invasion but did not alter proliferation and apoptosis. Meanwhile, the growth of peritoneal nodules is inhibited and survival is prolonged in mice administrated with siSYT13 intraperitoneally (42). Further analysis revealed that SYT13 expression was correlated with shorter peritoneal recurrence-free survival and OS. Multivariate analysis demonstrated that SYT13 positivity in lavage fluid was a vital prognostic factor for predicting GC peritoneal recurrence (*P* = 0.0246, HR = 3.69, 95% CI = 1.18–12.74) (43). Kanda et al. constructed a mouse xenograft model with GC peritoneal metastasis and discovered that intraperitoneal administration of amido-bridged nucleic acid-modified anti-SYT13 antisense oligonucleotides could inhibit the formation of peritoneal metastatic nodules and significantly prolong survival (44).

SYT13 was overexpressed in CRC samples compared with the adjacent normal samples. *In vitro* experiments showed that silencing of SYT13 could depress the activity of the CRC cell lines RKO and HCT116, including proliferation, colony formation, invasion, and migration. *In vivo* assays also showed the role of SYT13 in promoting tumor growth. In addition, after SYT13 knockdown, N-cadherin, Vimentin, and Snail expression were all inhibited, suggesting that downregulation of SYT13 may inhibit the occurrence of EMT (45). Keck et al. analyzed RNA isolated from matched primary neuroendocrine tumors of the small bowel (SBNETs), liver metastases, and normal small bowel tissue in 12 patients by utilizing RNA-Seq and whole transcriptome expression microarrays (46). The results showed that SYT13 was overexpressed in tumorous tissues and was associated with the progression of SBNETs. However, more laboratory and clinical investigations are warranted.

Downregulation of SYT13 in the LUAD cell lines A549 and H1299 could successfully suppress cell proliferation and clonal activity, but enhance apoptosis. Moreover, the knockdown of SYT13 decreased the migration capacity of H1299 cells. These results demonstrated that SYT13 was a vital promoter in the development of LUAD (47). Furthermore, SYT13 is overexpressed in both clinical specimens and cell lines of estrogen receptor (ER)-positive breast cancer. SYT13 was also revealed to be positively correlated with several oncogenes predominantly expressed in ER-positive breast cancer by PCR array analysis (48). These results suggest that SYT13 has a positive correlation with ER-related signaling pathways in breast cancer.

In conclusion, SYT13 may serve as an oncogene in GC, CRC, SBNETs, LUAD, and ER-positive breast cancer. High expression of SYT13 predicted poor prognosis in GC. More investigations of the mechanism underlying the biological activity of SYT13 in these cancers are warranted.

### SYT14

Aberrant SYT14 was associated with neurodevelopmental abnormalities (49) and psychomotor retardation (50). Sheng et al. knocked down the expression of SYT14 in the human glioma cell line U87MG *via* RNAi, resulting in significant inhibition of cell proliferation and colony formation but a modest promotion of apoptosis. In parallel, more G2/M phase cells and fewer S phase cells were observed (51). These results reveal that SYT14 is upregulated in glioma cells and may participate in the occurrence and development of glioma.

### SYT16

SYT16 lacks calcium-sensing as well as a transmembrane domain (52). Bioinformatic analysis of TCGA database revealed that the expression levels of SYT16 in glioma samples were significantly lower than that in normal samples. Moreover, SYT16 was only expressed in grade II and grade III glioma and was positively correlated with tumor grade. The higher the histological grade, the lower the expression. Multivariate analysis showed that SYT16 was a significant prognostic factor for glioma (53). Nevertheless, more laboratory studies should be conducted to further validate the biological activity of SYT16.

### Other isoforms

Through bioinformatics analysis in online databases, the expression levels of SYT4, SYT9, and SYT14 were found to be upregulated in GC tissues compared with normal tissues and were negatively correlated with their methylation levels. Both the hypomethylation levels of SYT4, SYT9, and SYT14 and their high expression contributed to the suboptimal OS and DFS in GC. The expression of these three isoforms also played a key role in immune cell infiltration in GC (54). These findings suggested that these three isoforms might be reliable prognostic indicators and potential immunotherapeutic targets in GC patients.

## Future prospects

Although the results from previous studies appear to be promising, supporting evidence of SYTs as a tumor marker is still lacking since most studies were retrospective. In the future, large-scale prospective studies are needed to further assess the value of SYTs in the diagnosis and prognostic prediction. We suggest the following for further research: (1) At present, we still do not understand the mechanism by which SYTs affect the occurrence and development of cancer. More work is required to elucidate the proteins and pathways that interact with SYTs to promote tumor growth and metastasis for further understanding of the biological functions of SYTs in cancers. (2) With the continuous improvement of public databases, more and more tumor markers or potential prognostic biomarkers have been discovered (55–58). For example, many SYTs were found significantly associated with poor prognosis in GC when analyzed using the Kaplan–Meier plotter (KM plotter) database (Supplementary Figure 2). However, the vast majority of studies in cancer are focused on SYT 7, 8, 12, and 13 currently. Thus, the following studies could be further extended to other isoforms in the future. (3) In addition, more than two isoforms of SYT expression abnormalities have been found in some tumors. For example, the expression of SYT7, SYT8, and SYT13 is increased in GC tissues, and all three isoforms can promote the proliferation, migration, and invasion of GC cells (13, 34, 42). Whether there is an intrinsic link between them also requires further research. (4) The classical function of SYTs mainly relies on their interaction with Ca^2+^ *via* the C2 domain. Is this Ca^2+^ sensing activity of SYTs involved in cancer? Do SYTs affect cancer *via* Ca^2+^ binding or independent of Ca^2+^ binding? In-depth studies of these issues can give us a better understanding of the anti-cancer mechanism of SYTs. (5) To develop SYT-targeted therapy, the dose, route, frequency, and duration of administration of siSYTs or inhibitors should be optimized. In addition, the existence of a synergistic effect of siSYTs in combination with existing chemotherapeutic agents also needs to be confirmed. Through the above research, we will obtain a better understanding of the functions of the SYTs and may find a new target for anticancer therapy.

## Conclusion

In the past 5 years, an increasing number of studies have demonstrated that SYTs play important roles in the occurrence and development of cancers. At present, most studies have shown that SYTs serve as oncogenes after overexpression. Downregulation of SYTs can inhibit the proliferation, migration, and invasion of cancer cells, but promote apoptosis. Through more in-depth research, SYTs may become a new target for the treatment of tumor diseases in the future.

## Author contributions

HS and NX searched the literature for recent advances in the field. HS wrote the manuscript. KW designed the study and edited and revised the manuscript. All authors approved the final version to be published.

## Supplementary material

Supplementary Figure 1 Synaptotagmins currently known to play an important role in the occurrence and development of cancers. All these SYTs in this figure play as oncogenes. SYTs, synaptotagmins; NSCLC, non-small cell lung cancer; HNSCC, head and neck squamous cell carcinoma; OSCC, oral squamous cell carcinoma; LUAD, lung adenocarcinoma; PTC, papillary thyroid cancer.

Supplementary Figure 2 Correlation between the expression levels of SYTs and patient survival. Expression data were analyzed using KM plotter (http://kmplot.com/). Patients were split by median expression. HRs (hazardous ratios), 95% CIs (confidence intervals), and log-rank *P*-values are indicated. **(A)** The relationship between overall survival and expression levels of SYTs in gastric cancer patients. **(B)** The relationship between relapse-free survival and expression levels of SYTs in gastric cancer patients.
