# Supplementary figures and images for: Potential roles of synaptotagmin family members in cancers: Recent advances and prospects
Source: Front Med (Lausanne). 2022 Aug 8;9:968081. doi: 10.3389/fmed.2022.968081 (PMC9393329; doi:10.3389/fmed.2022.968081)

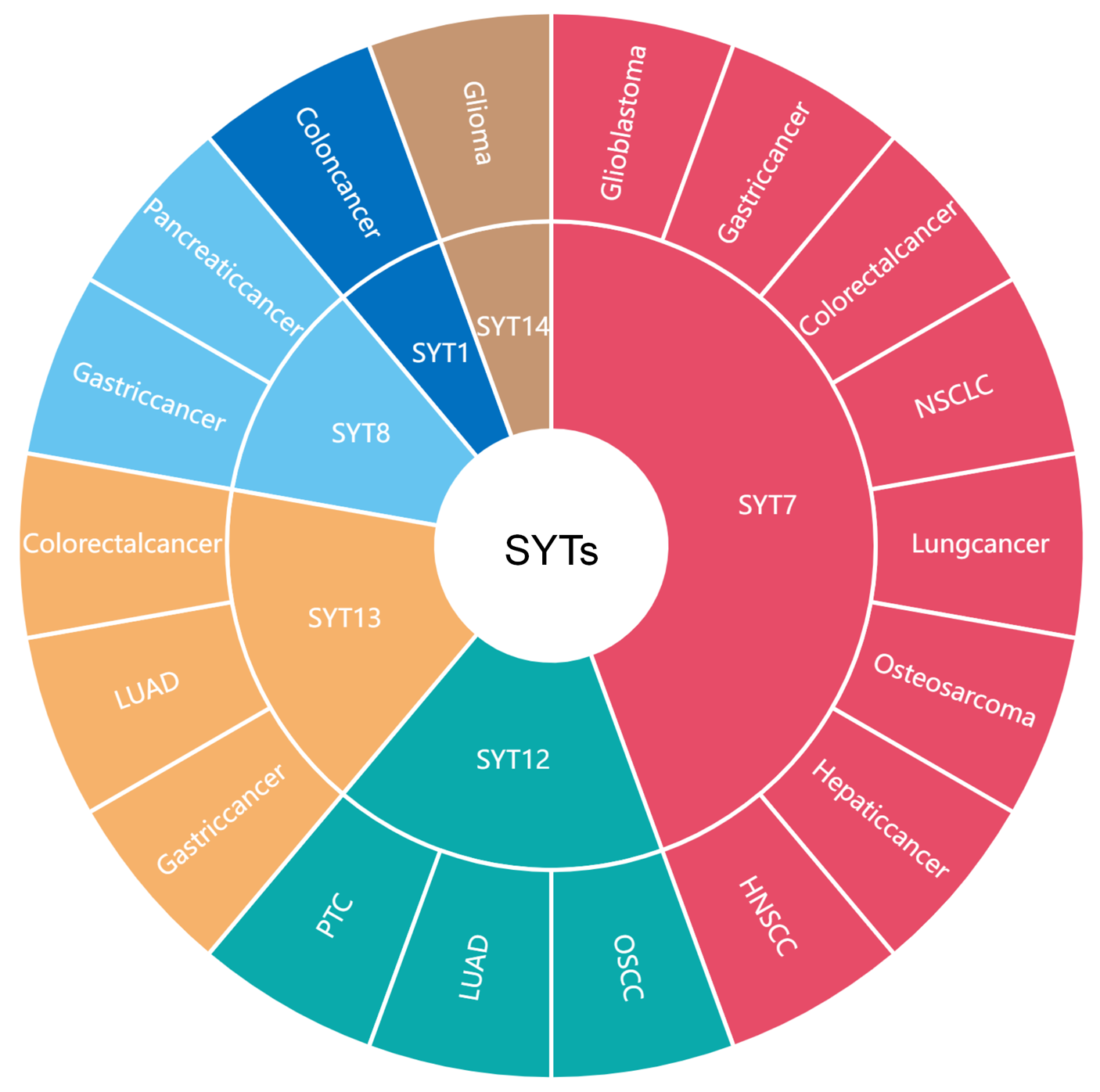

Supplement: Supplementary Figure 1 — Synaptotagmins currently known to play an important role in the occurrence and development of cancers. All these SYTs in this figure play as oncogenes. SYTs, synaptotagmins; NSCLC, non-small cell lung cancer; HNSCC, head and neck squamous cell carcinoma; OSCC, oral squamous cell carcinoma; LUAD, lung adenocarcinoma; PTC, papillary thyroid cancer. [file Image_1.TIF]

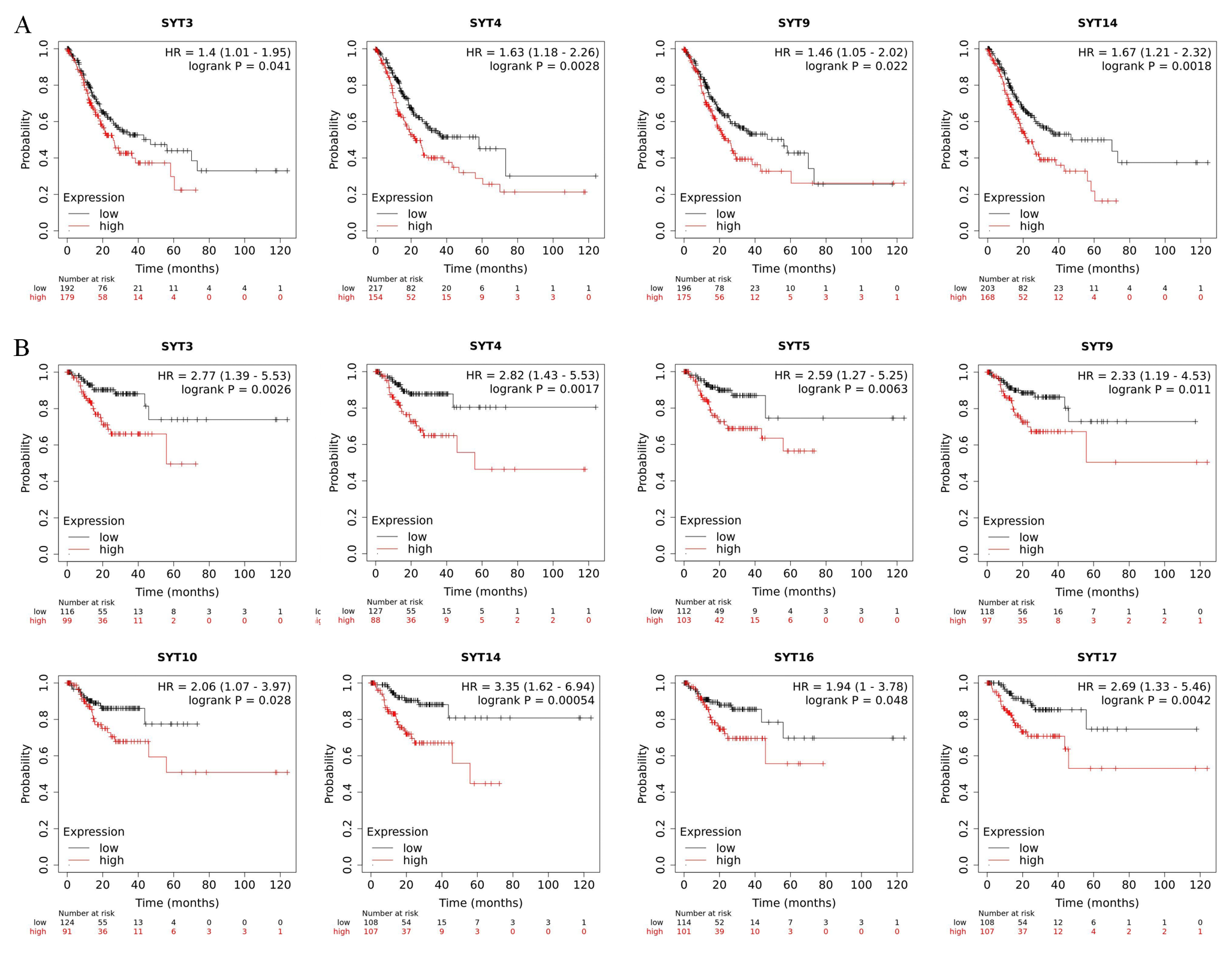

Supplement: Supplementary Figure 2 — Correlation between the expression levels of SYTs and patient survival. Expression data were analyzed using KM plotter (http://kmplot.com/). Patients were split by median expression. HRs (hazardous ratios), 95% CIs (confidence intervals), and log-rank P-values are indicated. (A) The relationship between overall survival and expression levels of SYTs in gastric cancer patients. (B) The relationship between relapse-free survival and expression levels of SYTs in gastric cancer patients. [file Image_2.TIF]
